# International Response under the Antarctic Treaty System to the Establishment of A Non-native Fly in Antarctica

**DOI:** 10.1007/s00267-021-01464-z

**Published:** 2021-04-15

**Authors:** Mónica Remedios-De León, Kevin Andrew Hughes, Enrique Morelli, Peter Convey

**Affiliations:** 1 Entomology Section, Facultad de Ciencias, Universidad de la República, Montevideo, Uruguay; 2 British Antarctic Survey, Natural Environment Research Council, High Cross, Madingley Road, Cambridge, CB3 0ET UK

**Keywords:** Invasive, Alien, Eradication, Antarctic Treaty, Committee for Environmental Protection

## Abstract

Antarctica currently has few non-native species, compared to other regions of the planet, due to the continent’s isolation, extreme climatic conditions and the lack of habitat. However, human activity, particularly the activities of national government operators and tourism, increasingly contributes to the risk of non-native species transfer and establishment. *Trichocera (Saltitrichocera) maculipennis* Meigen, 1888 (Diptera, Trichoceridae) is a non-native fly originating from the Northern Hemisphere that was unintentionally introduced to King George Island in the maritime Antarctic South Shetland Islands around 15 years ago, since when it has been reported within or in the vicinity of several research stations. It is not explicitly confirmed that *T. maculipennis* has established in the natural environment, but life-history characteristics make this likely, thereby making potential eradication or control a challenge. Antarctic Treaty Parties active in the region are developing a coordinated and expanding international response to monitor and control *T. maculipennis* within and around stations in the affected area. However, there remains no overarching non-native invasive species management plan for the island or the wider maritime Antarctic region (which shares similar environmental conditions and habitats to those of King George Island). Here we present some options towards the development of such a plan. We recommend the development of (1) clear mechanisms for the timely coordination of response activities by multiple Parties operating in the vicinity of the introduction location and (2) policy guidance on acceptable levels of environmental impacts resulting from eradication attempts in the natural environment, including the use of pesticides.

## Introduction

Many environments on the planet have been affected by non-native invasive species, including the islands surrounding Antarctica and the fringes of the continent itself (Frenot et al. 2005; Convey and Lebouvier 2009; Hughes et al. 2015; Pyšek et al. 2020). The spatial isolation of the Antarctic continent, its extreme weather conditions and the very small extent of appropriate habitat offer some protection against colonization by non-native species (Hughes and Convey 2010). Within the Antarctic Treaty area (the area south of latitude 60° S) human activities, particularly those of tourism and national government operators, increasingly contribute to the risk of non-native species being transported to the continent along anthropogenic pathways (Frenot et al. 2005; Whinam et al. 2005; Hughes et al. 2005, 2011; Convey et al. 2012; Lee and Chown 2009; Hughes and Convey 2010; IAATO 2015). The South Shetland Islands, located north-west of the Antarctic Peninsula, have been identified as the region of Antarctica most at risk from non-native species introductions, due to a combination of high human activity levels, relatively benign climatic conditions compared to other Antarctic regions, and predicted climate change impacts, with species being transported to the region from countries across the planet (Chown et al. 2012; Huiskes et al. 2014; Convey and Peck 2019; Hughes et al. 2020).

Many native Antarctic organisms can survive extreme climatic conditions, and have been described as ‘stress selected’, investing considerable resources in stress tolerance strategies (Convey 1996). Biological interactions, including competition and predation, have generally been regarded as insignificant in Antarctic terrestrial ecosystems and competitive ability does not form part of stress-selected life-history strategies (Convey 1996; Hogg et al. 2006), although recent research suggests a combination of abiotic and biotic factors may influence invertebrate abundance in some circumstances (Caruso et al. 2013; Potts et al. 2020). It is increasingly clear on some sub-Antarctic islands that non-native invasive predators (carabid beetles) have strongly negative impacts on many, often endemic, members of the native terrestrial invertebrate communities, which do not include analogous functional guilds (e.g. Lebouvier et al. 2020). Such species can be considered ecosystem engineers. In an analogous fashion, non-native detritivorous insects introduced to sub- and maritime Antarctic islands have been shown to have a substantial impact on rates of decomposition in specific shoreline (Hänel and Chown 1998) and moss peat habitats (Hughes et al. 2013), increasing these by approaching an order of magnitude, relative to the entire native invertebrate communities of these habitats. Chronically low rates of decomposition are a major limitation on terrestrial ecosystem processes in the continental and maritime Antarctic (Convey et al. 2014). Non-native invasive species globally are generally strong competitors and any non-native taxa that are introduced to and able to survive in Antarctica are likely to face little resistance from the native biota. Consequently, should a non-native species become established outside the man-made facilities present in Antarctica, the risk of this species becoming more widely established in the natural environment and potentially invasive is increased. If this happens, it can have serious impacts on Antarctic species and ecosystems (e.g. Hughes et al. 2013; Molina-Montenegro et al. 2019; Lebouvier et al. 2020).

## Trichocera: *Native Range, Life-history and Physiological Characteristics*

Members of the dipteran genus *Trichocera* are widely distributed in the Northern Hemisphere, especially in boreal regions (Dahl and Alexander 1976; Dahl and Krzemińska 1997). Members of the genus are tolerant of both warm and cold climatic conditions, but in higher latitude locations often exist synanthropically (for a recent review see Potocka and Krzemińska 2018).

*Trichocera (Saltitrichocera) maculipennis* Meigen, 1888 (Diptera, Trichoceridae) is a non-native fly recently introduced into the maritime Antarctic South Shetland Islands (Volonterio et al. 2013; Chown and Convey 2016; Potocka and Krzemińska 2018), as well as earlier to the sub-Antarctic Kerguelen Islands in the Indian sector of the Southern Ocean (Séguy 1940, 1953; Dahl 1966). The natural distribution of *T. maculipennis* extends from the Arctic to southern areas of the Mediterranean region (for an overview see Potocka and Krzemińska 2018). With its wide distribution in northern boreal regions, the species has life-history flexibility and physiological characteristics that are appropriate for its survival and establishment in typical conditions throughout the maritime Antarctic (Potocka and Krzemiska 2018). It has four larval stages and, in natural northern habitats, adults can emerge from the pupa through the snow layer and be active on the snow surface (Hågvar and Krzemińska 2008). In synanthropic situations, larvae have been found in nutrient-rich substrates, such as composting plant matter and excrement, and as pests of stored vegetables, and they can also survive within semi-liquid or liquid substrates including drainage chambers and sewage treatment plants (Karandikar 1931; Lindroth 1931; Dahl 1966, 1967; Volonterio et al. 2013).

All life stages of *Trichocera* species are susceptible to extreme cold or heat, as well as desiccation (Dahl 1967) and their temperature tolerance range is similar to the short-term exposure survival envelopes documented for the two native Antarctic chironomid species *Belgica antarctica* and *Parochlus steinenii*, both of which also occur in the South Shetland Islands (reviewed by Convey and Block 1996; Chown and Convey 2016). *T. maculipennis* relies upon a low stable temperature regime to facilitate the development of the egg and larval life stages (e.g. see Plachter 1983). The duration of the life cycle depends largely on environmental conditions, but can take up to 1 year in their natural distribution, with adults emerging predominantly during the warmer months but also at other times depending upon the location (Hågvar and Krzemińska 2008). Summer monthly average field air temperatures throughout the maritime Antarctic are typically +1 to +3 °C, with the South Shetland Islands being one of the mildest parts of this region, and winter temperatures in habitats with a protective covering of snow remain consistently at high sub-zero levels (Convey et al. 2018) thus potentially presenting an ideal thermal environment for *T. maculipennis*.

## International Agreements on Non-native Species Management in Antarctica

The Antarctic Treaty area is under international governance through the Antarctic Treaty Consultative Meeting (ATCM), where the (currently) 29 Consultative Parties to the Antarctic Treaty make governance decisions through consensus. The Protocol on Environmental Protection to the Antarctic Treaty (also known as the Environmental Protocol or Madrid Protocol) sets out, in Annex II, strict rules concerning non-native species. Except for fresh foods, the introduction of any non-native species (including microorganisms) to the Treaty area is prohibited, unless a permit has been issued by an appropriate national government and measures are put in place to ensure the species’ subsequent eradication. Furthermore, Annex II states that any species not native to the Antarctic Treaty area that is introduced without a permit *‘*shall be removed or disposed of whenever feasible, unless the removal or disposal would result in a greater adverse environmental impact’. The Committee for Environmental Protection (CEP) was established by the Protocol to provide advice and formulate recommendations to the Parties in connection with the Protocol’s implementation. In recent years, the CEP has allocated the issue of non-native species introductions the highest priority in its 5-year work plan. Parties use the CEP as a forum to share information on environmental issues, including the management of non-native species. The CEP non-native species manual (CEP 2019) recommends that ‘to be effective, responses to introductions should be undertaken as a priority, to prevent an increase in the species’ distribution range and to make eradication simpler, cost-effective and more likely to succeed’. Therefore, the successful management of non-native species should be a priority.

In this paper, we examine the introduction and establishment of the non-native dipteran *T. maculipennis* to King George Island, South Shetland Islands, Antarctica. In the CEP XX Final Report (available at: https://documents.ats.aq/ATCM40/fr/ATCM40_fr001_e.pdf) the Committee encouraged Parties to ‘develop coordinated standardised monitoring and eradication programmes to effectively control the spread of the flies’. In an effort to advance this work, we go on to describe the international response to date, examine future options for the eradication or control of the species and recommend areas of policy development to facilitate future rapid management action.

## Methods

### Study Area

**Fig. 1:**
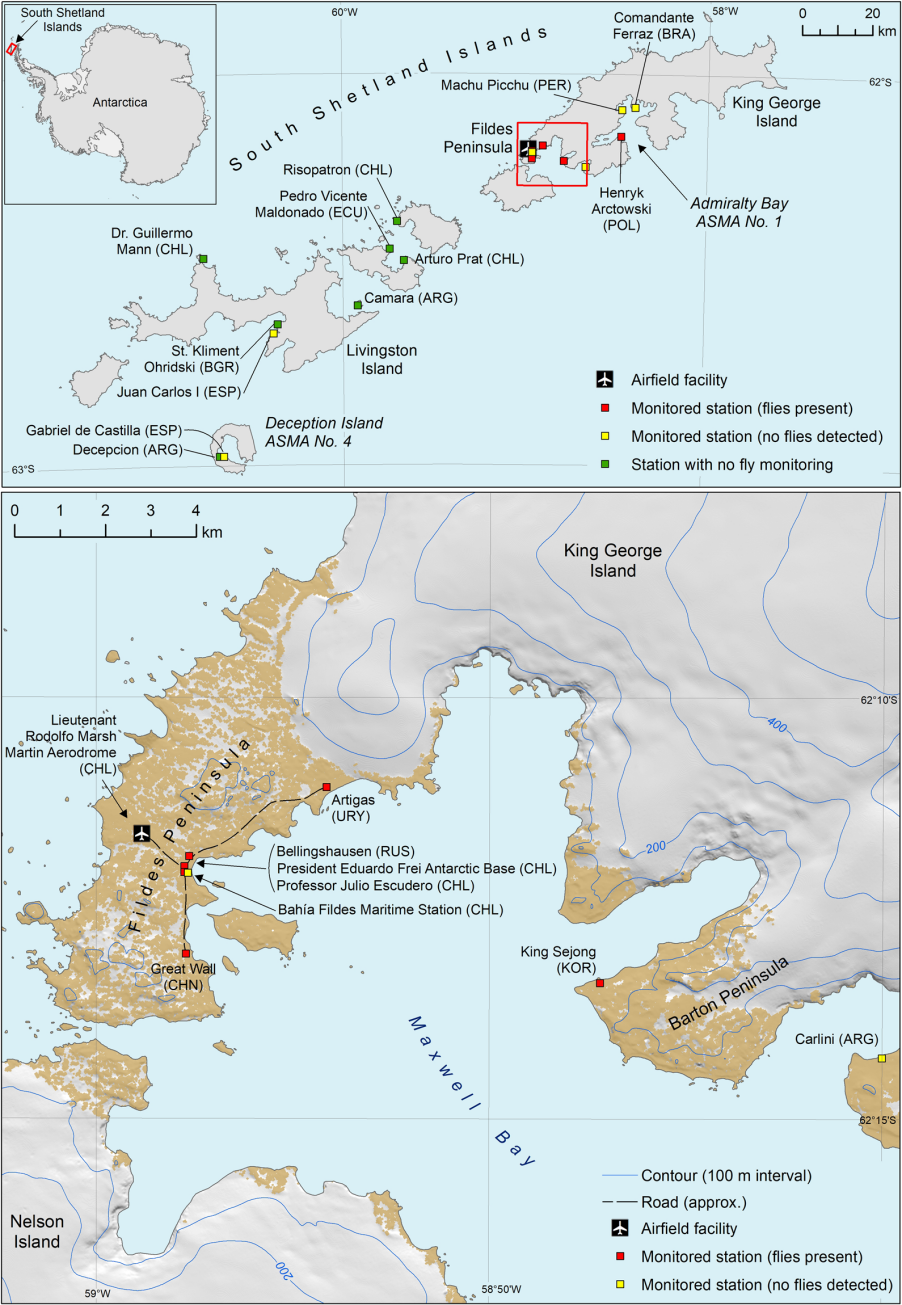
Map indicating the locations of research stations in the vicinity of the *Trichocera maculipennis* introduction area. The stations are classified as follows: stations undertaking or planning monitoring, where evidence of flies has been found (red squares), stations undertaking or planning monitoring with no evidence of fly colonisation (yellow squares) and stations not yet involved in a fly monitoring programme (green squares). It is not confirmed whether flies are present at Bahía Fildes Maritime Station as the monitoring data were lost in a fire during the 2017/18 season and sampling was not resumed for the 2018/19 season (see Uruguay et al. 2019). Flies have been observed within the loading area, bathrooms and waiting room of Lieutenant Rodolfo Marsh Martin Aerodrome (M. Remedios-De León, pers. obs.)

The extent of the current study area comprises the Antarctic research stations currently colonized by *T. maculipennis* on King George Island, South Shetland Islands, and the intervening ice-free ground. Other adjacent research stations and ice-free areas, including on neighbouring islands, are also in scope as there may be a risk of imminent colonization by the fly (see Fig. 1).

### Information Sources

Information on the management of non-native species that has been generated by the CEP and agreed by the Antarctic Treaty Parties was obtained from the CEP Non-native Species Manual Edition 2019 (CEP 2019).

Documents submitted by Parties to meetings of the CEP and the ATCM were obtained from the Antarctic Treaty Secretariat website (www.ats.aq).

## Results

### Current Distribution of *T. maculipennis* in Antarctica

*T. maculipennis* (Diptera, Trichoceridae) is a non-native fly recently introduced into the maritime Antarctic South Shetland Islands (Volonterio et al. 2013; Chown and Convey 2016; Potocka and Krzemiska 2018), north-west of the Antarctic Peninsula. To date, all observations of *T. maculipennis* in the South Shetland Islands have been on King George Island. The first record was of a specimen captured on a moss surface located close to Presidente Eduardo Frei Montalva station, on the Fildes Peninsula (Volonterio et al. 2013). Recent surveys indicate that the species is now present within seven research stations on the Fildes Peninsula, Barton Peninsula and around Admiralty Bay (i.e. Artigas (Uruguay), Bellingshausen (Russian Federation), Professor Julio Escudero (Chile), Presidente Eduardo Frei Montalva (Chile), Lieutenant Rodolfo Marsh Martin Aerodrome (Chile), Great Wall (China), King Sejong (Republic of Korea) and Arctowski (Poland) stations) (see Table 1 and Fig. 1). The fly has not been recorded from Comandante Ferraz (Brazil), Machu Picchu (Peru) or Carlini (Argentina) stations. The discovery, in October 2017, of larvae and adults in the station sewage system at Arctowski Station, Admiralty Bay, was particularly notable as the station is c. 20 km from Fildes Peninsula and separated by a glacier barrier (Potocka and Krzemińska 2018) (Fig. 1). It is not known if the species was transported from the Fildes Peninsula/Maxwell Bay area to Admiralty Bay by natural or anthropogenic mechanisms. While Bellingshausen, Frei and Escudero stations are located adjacent to one another, most other affected stations are several kilometres apart (see Fig. 1).

**Table 1:** Chronology of observations and reports of the non-native dipteran *T. maculipennis* on King George Island (KGI), South Shetland Islands, Antarctica

Location	Date	Notes	Reference
Near Presidente Eduardo Frei Montalva Station, Fildes Peninsula, KGI	2006	Free flying adult	P. Fretwell, British Antarctic Survey, pers. comm.
Artigas Station (Uruguay), Fildes Peninsula, KGI	2006 (?)	Early eradication attempt in 2008 unsuccessful. Species observed in the surrounding environment. The fly has recently been observed outside the station. Joint monitoring programme in place.	Volonterio et al. (2013); COMNAP (2019)
Frei Station (Chile), Fildes Peninsula, KGI	Pre-2009/2010 season	Identification to species level not complete. Larvae still persist in the sewage treatment plant.	V. Vallejos, pers. comm., quoted in Peter et al. (2008).
King Sejong Station (Korea) Barton Peninsula, KGI	2013/2014	Adults observed in station, no larvae observed. Eradication initiated in October 2015 using physical cleaning, pesticide and UV traps. The flies were detected again in December 2015	Republic of Korea et al. (2016)
Escudero Station (Chile), Fildes Peninsula, KGI	Jan 2015	Adults found around station windows, no larvae observed	P. Convey, pers. obs.; T. Contador, pers. comm.
Lake Uruguay, near Artigas Station (Uruguay)	Feb 2017	Adults seen in the natural environment	M. Remedios-De León, pers. obs.
Arctowski Station (Poland), Admiralty Bay, KGI	Oct 2017	Larvae and adults of *T. maculipennis* were found in the station sewage system	Potocka and Krzemińska (2018)
Great Wall Station (China)	2018/19	Very low numbers detected, indicating that a reproducing population may not be present on the station.	Uruguay et al. (2019)
Bellingshausen Station (Russian Federation), Fildes Peninsula, KGI	2018/19	Main swarm is observed at a nearby stream and around the sewage water tank.	COMNAP (2019)
Lieutenant Rodolfo Marsh Martin Aerodrome (Chile)	2019	Adult specimens were observed in bathrooms, the waiting room and loading area of the aerodrome.	M. Remedios-De León (pers. obs.)

Multiple unpublished and anecdotal observations have confirmed the presence of *T. maculipennis* in the natural environment beyond station buildings. The initial observations of an adult fly near Frei station, and others near the Uruguayan station, indicate that survival (and plausibly reproduction) are possible in local natural conditions beyond the confines of station buildings (Table 1; Volonterio et al. 2013). The observation and collection of adults (including gravid females) in the natural environment at Lake Uruguay, near Artigas Station (M. Remedios-De León, pers. obs.) shows their longer-term survival ability under maritime Antarctic summer conditions. Similarly, at Bellingshausen Station, females have been observed in the natural environment along the water channel outside the station buildings. To date, larvae have not been observed in the natural environment (outside mesocosm experiments, see below), although this may most likely reflect the fact that very few searches have been attempted.

Within stations, *T. maculipennis* has been observed predominantly in association with sewage storage or treatment facilities, consistent with earlier literature reporting synanthropic habitats occupied within its natural distribution. Large numbers of adults, larvae and pupae have been observed in the septic chambers of Bellingshausen and Artigas Stations. At Arctowski Station, adult flies were active and flying inside the sewage system, where temperatures under the lid of the sewage tank fluctuated between 1 and 3 °C, while the outside temperature was between −3.5 and −2 °C (Potocka and Krzemiska 2018). Such low positive temperatures are comfortably within the known survival range of *T. maculipenis* and provide an ideal environment for the species, as well as being entirely typical of summer air temperatures across the maritime Antarctic (Convey et al. 2018).

### International Collaborative Efforts to Deliver Effective Monitoring and Control

In 2016, the first survey ‘A short questionnaire on non-native flies in Antarctic stations' was carried out at stations on King George Island, as the first formal step towards developing a coordinated international response (see Attachment A in Republic of Korea et al. 2017; Table 2). Questions on the presence of the fly in buildings, facilities and the surrounding area of each station were included in the survey. Uruguay, the Republic of Korea, Chile and the Russian Federation then initiated a pilot monitoring programme on their stations to track and find methods to prevent the expansion of *T. maculipennis* on King George Island (Republic of Korea et al. 2017). The actions implemented included the installation of adhesive traps inside the station facilities of all four nations and pitfall traps in the external environment around the Uruguayan and Korean stations. The results of the joint monitoring programme of *T. maculipennis* populations at the King Sejong and Artigas stations during the 2017–2018 period were presented at the CEP XXII meeting in 2019 (Uruguay et al. 2019). In the 2018/19 summer season, Argentina, Brazil and China joined the monitoring programme, implementing monitoring at Carlini, Ferraz and Great Wall stations, respectively, while Peru commenced participation at Machu Picchu Station during the 2019/20 season. The Scientific Committee on Antarctic Research (SCAR), which provides impartial scientific advice to the CEP on environmental matters, recognised the potential risk presented by *T. maculipennis*, by awarding a SCAR Fellowship to Uruguayan researchers to develop management options for the species in King George Island in 2018 (Uruguay et al. 2019; see https://www.scar.org/awards/fellowships/overview/).

**Table 2:** Policy paper submissions to the Antarctic Treaty Consultative Meeting’s Committee for Environmental Protection on the introduction and management of *T. maculipennis* on King George Island (available at: www.ats.aq)

Year	Submitting CEP Members	Title	Reference
2011	UK and Uruguay	Colonisation status of known non-native species in the Antarctic terrestrial environment (updated 2011)	ATCM XXXIV IP50 (United Kingdom and Uruguay 2011)
2016	Republic of Korea, UK, Chile and Uruguay	Non-native flies in sewage treatment plants on King George Island, South Shetland Islands	ATCM XXXIX WP 52 (Republic of Korea et al. 2016)
2017	Republic of Korea, Uruguay, Chile and the UK	Inter-Parties’ Action Plan to Manage the Non-Native Flies in King George Island, South Shetland Islands	ATCM XL WP 26 (Republic of Korea et al. 2017)
2019	Uruguay, Argentina, Brazil, Chile, China, Germany, Republic of Korea, and the Russian Federation	Report of the 2018/2019 summer campaign of the joint monitoring programme of non-native flies in King George Island/Isla 25 de Mayo	ATCM XLII IP120 (Uruguay et al. 2019)
2019	Council of Managers of National Antarctic Programmes (COMNAP)	Report on the extent of sewage treatment plant infestations across the Antarctic Treaty area: Survey results	ATCM XLII IP38 (COMNAP 2019)

Adhesive insect traps are currently installed at Artigas, Bellingshausen, Carlini, Escudero, Ferraz, Great Wall and King Sejong stations. At the end of the 2018/19 summer season, ultraviolet light traps were installed within Artigas, Bellingshausen, Escudero and King Sejong stations to assess their effectiveness as a population control measure. The installation of these traps has substantially decreased the number of adults present and has proven the most effective control measure identified to date (Uruguay et al. 2019). Analyses using environmental DNA techniques have been initiated to assess the presence of *T. maculipennis* in the surroundings of stations on Barton and Fildes Peninsulas. The Korea Polar Research Institute (KOPRI) has carried out molecular analyses to assess population relatedness between Escudero, Artigas and King Sejong stations. Preliminary results suggest that dispersal and subsequent establishment of the fly have taken place between these stations on King George Island, rather than the species being present as a result of multiple separate introduction events (Uruguay et al. 2019). KOPRI also examined development of *T. maculipennis* eggs and larvae under laboratory conditions and in mesocosms within a lake near King Sejong Station. In both environments, the eggs hatched and developed to larvae, showing that the different life stages can survive and complete at least part of the life cycle under natural local environmental conditions (Uruguay et al. 2019).

Uruguay and the Republic of Korea have made unilateral attempts to eradicate *T. maculipennis* from their respective stations on King George Island. In 2008, an eradication effort at the Uruguayan station was made involving the use of permethrin pesticide and sewage system cleaning. Initially, the effort was believed to have been successful with no further observations of the fly for over 2 years. However, in 2011, individuals were again found within the station buildings, indicating either that the eradication effort had not been fully effective or that re-colonization of the station had occurred by individuals from the surrounding natural ecosystem or other local synanthropic populations (Volonterio et al. 2013).

Following a similar pattern, once the presence of the species had been reported at the Korean King Sejong Station, cleaning of the wastewater facilities at the station was carried out in 2015 in an eradication attempt (Republic of Korea et al. 2016). However, although the use of pesticides and UV traps appeared to partially control the *T. maculipennis* population here, eradication was not achieved, possibly due to the limitations of these methods for effective elimination of eggs and early-stage larvae that may remain in place within the sewage system. Korea suspended the use of pesticides in early 2018, through concern that they could induce resistance in the fly species.

### Options for Further Action

Consensus has been established on the need for continuous exchange of information and monitoring among CEP members representing Parties that operate year-round stations on King George Island (Uruguay et al. 2019). However, at present, a management plan for *T. maculipennis* on King George Island (and beyond) has not been developed. Here we describe practical considerations to inform the development of such a plan, building on measures recommended by COMNAP, SCAR and the CEP (see also Table 3). Several possible response options and actions exist:

**Table 3:** Recommended practical control measures to reduce the population size and potential further distribution of *T. maculipennis*

No.	Measures
	Education of station personnel and those arriving on King George Island
1	Implementation of effective educational and training practices. Posters and information leaflets should be placed at the entry points to Antarctica and at each station on King George Island informing visitors of the presence of the invasive species and the efforts that are being made to eradicate it from Antarctica. Educational information should also be disseminated at Punta Arenas airport and other points of entry from the South American mainland.
2	Station personnel should be made aware of the importance of cleanliness of rooms and common spaces to ensure locations for flies to shelter or reproduce are minimized.
	Monitoring
3	Monitoring activities should be put in place, or existing monitoring maintained, across the stations on King George Island, and potentially beyond. To quantify fly numbers, sticky traps and ultraviolet traps should be deployed in potential breeding areas, with these methods also having the benefit of reducing flying adult population numbers.
4	To track the potential spread of *T. maculipennis*, monitoring should also be undertaken at research stations and in the natural environment, in areas beyond the known distribution of the fly.
5	To ascertain the environmental requirements of *T. maculipennis* in Antarctica, wastewater treatment chamber and field environmental temperatures should be recorded. This would allow a comparison of temperature vs. survival rates, thereby informing management practices applied to the sewage system that would make them less favourable for reproduction and survival of the species.
	Reporting
6	Personnel on stations in the South Shetland Islands should report immediately the presence of flies on station or in the natural environment to their station leader and those responsible for environmental management and protection. Steps should be taken to minimize the likelihood of inadvertent dispersal of the fly to other locations/buildings.
	Steps to reduce dispersal of the flies from colonized stations
7	Sewage systems should be airtight or, failing that, be supplied with a fine mesh grid to prevent the movement of adult flies. Grills should be placed in ventilation ducts to prevent the entry and exit of adult flies. Frequent cleaning of the sewage systems should be considered, for instance monthly.
8	Storage areas for materials under buildings should be removed to reduce the availability of shelter locations for adult flies.
9	To prevent dispersal of the flies, vehicles entering and leaving stations should be rigorously cleaned, which may require a dedicated cleaning location.
	Steps to prevent re-introduction of *T. maculipennis* *or introduction of other invertebrates from outside Antarctica*
11	Biosecurity measures should be implemented by all national Antarctic programmes and the tourism industry to ensure the risk of non-native species introductions is minimized. Biosecurity guidance and information can be obtained from the CEP *Non-native Species Manual* (Edition 2019) and the SCAR and COMNAP *Inter‐continental checklists for supply chain managers of the national Antarctic programmes for the reduction in risk of transfer of non‐native species* (version May 2019).
	International cooperation and coordination
12	National Antarctic programmes should meet (either physically or virtually) at least annually to review progress in addressing the fly introduction and to plan further action.
13	Science: National Antarctic programmes should continue to work together in a coordinated manner, using comparable methodologies to monitor fly population numbers, and sharing scientific information.
14	Environmental management: National operators should develop common methodologies to control the fly and reduce dispersal through inter-station movement.
	Eradication
15	Earlier experiences at Artigas and King Sejong stations have shown that unilateral eradication of *T. maculipennis* from research stations results in rapid re-colonization within a few weeks/months. Stations where the fly has been eradicated may be rapidly recolonized from populations resident in other stations or in the natural environment. Therefore, it is essential that national Antarctic progammes coordinate their eradication activities so that all populations within stations are eradicated simultaneously, thereby reducing the opportunity for re-colonization.

#### Do nothing

Faced with the presence of the species within an increasing number of research stations on King George Island, and increasing reports from the natural environment, the Parties involved could decide not to take any control/eradication measures. However, such a response would be contrary to Annex II to the Environmental Protocol. It is clearly evident from actions taken to date that the Parties concerned have chosen not to adopt this option.

#### Ongoing monitoring and local control

Parties on King George Island have already taken considerable steps in monitoring the presence and numbers of flies within several research stations. However, scope exists for survey and monitoring to commence at locations beyond the stations themselves and at a greater distance from those already colonized. For example, the potential exists to rapidly transport flies by aircraft from Fildes Peninsula to other distant research stations, for example, the UK’s Rothera Research Station 750 km to the south, and other regularly used field locations accessed by air from King George Island, making monitoring at such locations essential. With this in mind, the observation of *T. maculipennis* in bathrooms, the waiting room and loading area of Lieutenant Rodolfo Marsh Martin Aerodrome (M. Remedios-De León, pers. obs.) is a major cause for concern and immediate steps should be taken to control the fly’s presence here and reduce the risk of dispersal via aircraft. Natural dispersal mechanisms, including wind, may also lead to dispersal of the fly to other islands in the South Shetland Islands archipelago, to the Antarctic Peninsula only 80–100 km to the south-east, or even to the more distant South Orkney Islands 700 km away but for which air mass trajectory modelling demonstrates the plausibility of reasonably frequent transfer within 24 h or less (Biersma et al. 2018).

If monitoring confirms colonization of this species in natural ecosystems, with gravid females and viable immature stages present, considering the pre-adapted features of the biology of the species the likelihood of any eradication attempt being successful may be low, and the impact of this detritivore on the fundamentally important and currently limiting ecosystem process of decomposition may be considerable. Maintaining ongoing monitoring as a tool to evaluate and identify any changes in populations of native and non-native terrestrial invertebrates is therefore essential. This monitoring should be implemented, at a minimum, with regular inspection of traps installed at all stations present on King George Island and in the adjacent natural environment. Polish researchers have developed methods for the molecular identification of *T. maculipennis* (Potocka et al. 2020). While adult flies can be easily identified based upon their morphological characteristics, molecular methodologies could provide a means of rapid identification of the other life cycle stages. Local control measures should continue to be implemented, both to reduce existing populations and to eradicate new ones, in as comprehensive a manner as possible at all affected locations. Parties involved in the monitoring should alert scientists and logistic coordinators in the wider Antarctic community of any new records. Monitoring will also have utility in identifying the presence of other non-native species, bearing in mind no such long-term monitoring programme has been put in place to date.

#### Action to prevent wider establishment in the natural environment

The biological characteristics of *T. maculipennis* make further dispersal by natural or assisted means and colonization of other Antarctic ecosystems likely, especially given the overall environmental similarity across much of the western Antarctic Peninsula region (Hughes et al. 2010; Convey et al. 2018; Bartlett et al. 2019; Pertierra et al. 2019). The species may use air currents to aid dispersal and thereby travel significant distances. It has not yet been possible to verify such movement of adults as, so far, only females have been recorded in low numbers in natural ecosystems beyond the confines of stations. Further, given that sewage systems can host substantial reproducing populations of the fly, their designs should be modified to ensure that any flies within already contaminated facilities are not able to gain access to the natural environment.

#### Minimize anthropogenic facilitation of movement of *T. maculipennis* to new locations in the South Shetland Islands, or further afield

Adult non-native flies could be transported to other locations in association with cargo or on aircraft or ships (Hughes et al. 2019), where *T. maculipennis* may colonize previously unaffected research stations or the natural environment. This makes the identification of locations with a high risk of establishment an essential step to implement monitoring and control practices efficiently. National operator, tourist and commercial fisheries operations must have a high awareness of this risk. It is unknown if the larvae can be transported in clothing/footwear or cargo, since no attempts have been made to quantify them in the field. However, it is appropriate to note that the potential of such dispersal routes for larvae of the invading and also detritivorous chironomid midge *Eretmoptera murphyi* on Signy Island (South Orkney Islands) has been identified as one of the most important risks (Bartlett et al. 2019). Clear and consistently applied biosecurity procedures are required for routes within and beyond the South Shetland Islands. Preventative measures to reduce the risk of introduction or re-introduction should include specific cleaning requirements for clothing, equipment and vehicles before operating in Antarctic regions, with effective training and audit processes to ensure these are carried out (cf. Hughes et al. 2010).

Responses should also include improved awareness and education. The potentially significant impacts on the key Antarctic ecosystem process of decomposition, should *T. maculipennis* become invasive, and the need for precautions to prevent transport of the fly must be communicated to all operators and visitors. Educational information could be provided at logistical hubs, in tourist reception points, vessels and camps, and in national operators’ stations, vessels and aircraft.

#### Attempt full eradication

This procedure could be developed in the context of two scenarios, the first if the flies are confirmed to be reproducing only within research stations, and the second where reproduction is taking place in the natural environment.

##### Eradication in and around research stations

Parties could propose an internationally agreed eradication protocol with effective control and monitoring measures that should be approved and executed by all affected stations simultaneously. Simultaneous application of control measures across all affected stations is essential to prevent re-colonization events from uncontrolled populations. In the absence of coordinated and simultaneous action, each colonized station would act as a source population for both further anthropogenically assisted dispersal and the potential invasion of the local natural environment and other stations.

The eradication protocol could take into consideration (as available) recorded annual population levels, breeding sites, duration of life cycles, population peaks and precise data on species dispersal. Logistic strategies should be developed, potentially involving the re-engineering of sewage systems and septic tanks, to allow initial and ongoing cleaning and maintenance of biosecurity precautions to prevent re-colonization, with such procedures applicable to both *T. maculipennis* and, with appropriate development, to any other analogous invertebrate introduction that occurs in the future. For example, the establishment of a closed-circuit system of ventilation could prevent/minimize the dispersal of insects into or from the external environment. Sufficient power to support the use of multiple ultraviolet light traps (the use of readily available battery-powered insect traps could also be considered as a new element of field survey activities remote from stations), as well as the use of sticky glue traps, during the whole year would help both to reduce and to monitor populations of reproductive adults. Any eradication attempt may have a higher chance of successful removal of the species from a given location if the colonized area is small (or only a few individuals are known to be present at the location), the necessary equipment is available to undertake the removal and a clear route for disposal of the material has been determined. Within stations, it would be essential to prevent the accumulation of waste where adult flies could find refuge (e.g. consider the installation of ultraviolet light traps in waste storage areas). Water supplied to stations could contain larvae and/or pupae, so consideration should be given to the use of appropriate filter systems, which would require frequent examination and replacement.

More generally, while the *T. maculipennis* introduction is an isolated incidence of apparently successful movement into the natural environment, there are already a number of cases of other invertebrates successfully establishing within station facilities (Hughes et al. 2005; Houghton et al. 2016; Bergstom et al. 2018). Thus, colonisation of research stations is an important potential intermediate step in an invasion, and it may be only a matter of probability or time until another species is introduced that has the ability to survive beyond the station itself.

##### Eradication from natural ecosystems away from stations

If the presence of reproductive females and viable immature stages in natural ecosystems is confirmed, eradication becomes considerably more challenging. One route could be the use of biological control strategies, such as the release in the field of populations of sterile males. This methodology has been used against other Diptera, such as *Cochliomyia hominivorax*, mosquitoes and tsetse flies (Marquez et al. 2019; Pan American Health Organization 2019), but would need considerable development for any given target species in Antarctica, particularly given the practical and environmental considerations. The Environmental Protocol prohibits the use of pesticides in the natural environment in Antarctica, but does allow their use for scientific, medical or hygiene purposes (see Annex III, Article 7). Therefore, under some circumstances, it may be possible to implement ‘in-situ' pesticide-based eradication strategies; interpretation of this element of the Protocol requires clarification.

## Discussion

In this paper, we have described the introduction of the non-native fly *T. maculipennis* to Antarctica, reported the increase in its distribution range and documented the efforts undertaken by National Antarctic Operators to eradicate it. We have also presented practical considerations to inform the development of a management plan for the fly on King George Island and beyond. However, the practicalities of non-native species management within the international Antarctic Treaty System present potential challenges that may benefit from further consideration by policymakers.

### ‘Multilateral’ vs. ‘Unilateral’ Action to Manage Non-native Species within the Antarctic Treaty Area

Most Treaty Parties, through their national operators, make unilateral decisions regarding the routine activities that are undertaken on their research stations and by their research teams within the Treaty area, although all activities should be undertaken only following an environmental impact assessment of the appropriate level (a process that also takes place through each operator’s national system). This approach has extended to responding to non-native species introductions. Most successful eradications of non-native plant species have been small-scale activities, generally with single or a low number of plants rapidly removed from a single location by researchers representing a single Treaty Party. Examples include the removal of *Puccinellia svalbardensis* from near Syowa Station by Japan (Tsujimoto et al. 2010), *Nassauvia magellanica* from Deception Island by the UK during a research visit supported by Spanish logistics (Smith and Richardson 2011; Hughes and Convey 2012), *Poa pratensis* from Cierva Point, Danco Coast (Pertierra et al. 2017), and *Poa annua* from Deception Island and close to three research stations on the Antarctic Peninsula by Chile (Molina-Montenegro et al. 2012) and from Signy Island by Italian researchers supported by UK logistics (Malfasi et al. 2019). Poland is in the midst of an ongoing attempt to eradicate *P. annua* in the vicinity of Arctowski Station and Antarctic Specially Protected Area (ASPA) No. 128 Western Shores of Admiralty Bay, on King George Island (Galera et al. 2017, 2019). No attempts have yet been made to eradicate invertebrates that have been established in the natural environment. Rather, all successful invertebrate eradications have involved populations reproducing within research station buildings. These include the dipteran *Lycoriella* sp. removed from Rothera Research Station (Hughes et al. 2005), the collembolan, *Xenylla* sp., eradicated from a hydroponic facility at Davis Station, East Antarctica (Bergstrom et al. 2018), Collembola removed from McMurdo Station hydroponic facility in 2003 and 2004, Acari eradicated from the South Pole Station hydroponic facility in 2006 and 2010 and successive treatment of infestations of the Scott Base hydroponic facility by Collembola between 2000 and 2005 (COMNAP 2014). However, some species persist synanthropically, for example, the dipteran *Lycoriella ingenue* that has persisted at Casey Station, Wilkes Land, for many years, despite several eradication attempts (Hughes et al. 2005; Houghton et al. 2016; COMNAP 2019).

Crucially, as a continent under multi-Party governance, decision-making on issues potentially affecting several Parties is generally more complex and time consuming compared to similar activities in areas under single-Party jurisdiction (i.e. sovereign nations). Should an established non-native species fail to be eradicated at an early stage, and disperse to multiple locations, management by a single Party will rapidly become impossible, and any response will inevitably need the attention, agreement and active engagement of all the Parties within the affected region to stand any chance of being effective (see Table 4). This may be problematic as, while Parties have been able to agree general (voluntary) measures to manage non-native species through the CEP, challenges may arise when there is a need for rapid international agreement on more responsive (and potentially costly) management of a specific non-native species on the ground using existing mechanisms of diplomacy involving multiple Parties (Hughes and Convey 2014). In one example, the persistence of a patch of the non-native grass *Poa pratensis* at Cierva Point, Danco Coast, since 1954 had been reported in the scientific literature on more than one occasion (e.g. Corte 1961; Smith 1996). When multiple Parties (Argentina, Spain and the UK) agreed to take coordinated action to address the introduction, it took over 3 years between studies ascertaining its then existing colonization status and the need for removal (Pertierra et al. 2013) and its subsequent eradication (Pertierra et al. 2017), with regular reporting to the CEP. In the case of the *T. maculipennis* on King George Island, unilateral action by Parties to address the introduction through cleaning of sewage systems necessarily developed into a coordinated multi-Party activity following the submission of a policy paper to CEP in 2016 (Republic of Korea et al. 2016). So far, the willingness of the Parties with facilities on the island to act in coordination is greatly encouraging. Spain, which has stations on other islands in the South Shetlands Islands group (i.e. Juan Carlos Station on Livingston Island and Gabriel de Castilla on Deception Island) has also indicated its willingness to participate in the future (A. Quesada, pers. comm.). Germany and the UK, while not operating major stations in the region, have long-standing scientific interests and are also following developments closely (Uruguay et al. 2019). Nevertheless, 15 years have elapsed since *T. maculipennis* was first observed on King George Island and Parties are still at the early stages of management of the introduced non-native species, which has itself expanded in distribution considerably.

**Table 4:** Breadth of national/international management response in Antarctica under different introduction scenarios

	Non-native species introduction type	Introduction location	Management response action^a^			Example references
			Unilateral	Parties in region	Many/all Parties	
1	Single or small number of plants within a limited area	Near an isolated station^b^	Yes	–	–	Pertierra et al. 2013, 2017; Tsujimoto et al. 2010; Molina-Montenegro et al. 2012
		Close to several research stations^c^	Yes	As appropriate	–	Peter et al. 2008; Smith and Richardson 2011
		Distant from any research stations^d^	Yes	As appropriate	–	–
2	Several plants spread across a wider area, potentially with seed bank present	Near an isolated station	Yes	–	–	–
		Close to several research stations	Yes	As appropriate	–	Galera et al. 2017, 2019
		Distant from any research stations	–	Yes	–	–
3	Invertebrates living synanthropically within a research station	Within an isolated station	Yes	–	–	Hughes et al. 2005; Bergstrom et al. 2018
		Within one station close to other research stations	Yes	As appropriate	–	COMNAP 2014
		Within several stations located in the same vicinity	–	Yes	As appropriate	Volonterio et al. 2013; Potocka and Krzemińska 2018; This study
4	Invertebrates living within the natural environment	Near an isolated station	Yes	As appropriate	As appropriate	Hughes et al. 2017
		Close to several research stations	–	Yes	As appropriate	Enriquez et al. 2019
		Distant from any research station	As appropriate	As appropriate	As appropriate	–
5	Terrestrial vertebrates (e.g. rodents)	Within or close to an isolated station	Yes	–	–	–
		Within or close to one station near other research stations	–	Yes	–	Peter et al. 2008
		Within or in the local area of several stations located in the same vicinity	–	Yes	–	–
6	Marine plants and invertebrates	Near an isolated station	?^e^	?	?	
		Close to several research stations	?^e^	?	?	Cárdenas et al. 2020
		Distant from any research station	?^e^	?	?	
7	Wildlife pathogen causing animal mass mortality events	Near an isolated station	–	Yes^f^	–	Leotta et al. 2006; Clarke and Kerry 1993
		Close to several research stations	–	Yes^f^	–	–
		Remote from any research stations	–	–	Yes^f^	Laws and Taylor 1957

^a^While all Parties in the vicinity of the introduction location may not be actively engaged in management of the introduced species, it would be appropriate to ensure Parties are kept informed of developments in case the situation escalates and other Parties need to become involved (see Hughes and Pertierra, 2016). Any management actions should be in addition to ongoing routine biosecurity measures that should be implemented by all national Antarctic programmes and the tourism industry, and as advocated by CEP, SCAR, COMNAP and IAATO. It may be difficult or impossible to ascertain which (if any) Party was responsible for a specific non-native species introduction, so all Parties should be prepared to engage in any response action, as necessary

^b^Examples of introductions near isolated research stations may include those located on islands or remote areas, for example, >50 km from other stations

^c^Station located within an cluster of stations, e.g., those on King George Island, Livingston Island, Larsemann Hills or Ross Island (McMurdo Sound)

^d^Species confirmed to be non-native and located far from existing station infrastructure, but potentially near a tourist visitor site, protected area or deep field research location

^e^As yet, there have been no attempts to eradicate marine non-native species within the Antarctic Treaty area, and this is likely to be almost impossible given the environmental conditions and available infrastructure and technologies (see McCarthy et al. 2019; Cárdenas et al. 2020; Hughes et al. 2020). Response action may be limited to communication of the introduction to other Parties operating in the region

^f^Response action in the event of an animal mass mortality event may be limited to the application of appropriate biosecurity measures, with communication of the event to all national operators and the tourism industry, including IAATO (CEP 2019)

### Opportunities to Enhance International Cooperation

The Environmental Protocol provides several management tools to help deliver agreed management practices. Antarctic Specially Managed Areas (ASMAs) are designated to assist in the planning and coordination of activities, avoid possible conflicts, improve cooperation between Parties or minimize environmental impacts. ASMAs have yet to be used to their full potential to help manage non-native species, with, for example, little or no practical advice on the management of the highly invasive non-native grass, *Poa annua*, provided within the management plan of ASMA No. 1. Admiralty Bay, King George Island (Molina-Montenegro et al. 2019). It remains to be seen if the management of *T. maculipennis* will be incorporated into the next version of this management plan. The Fildes Peninsula area of King George Island contains several research stations, an airstrip and hosts considerable tourism activity, yet it has not been designated as an ASMA, despite calls to do so (Braun et al. 2012; Convey 2020). Nevertheless, the ASMA tool, and the associated international ASMA management group, may provide one mechanism to communicate and deliver a coordinated international response to non-native species introductions in the area.

In locations where management groups that are formally endorsed by the ATCM have not been established, other mechanisms for communication and coordination may be employed. Korea, Chile, the UK and Uruguay recommended that the CEP ask COMNAP to play a central role in sharing information and best practices between Parties and other stakeholders concerning the *T. maculipennis* introduction (Republic of Korea et al. 2017). Furthermore, the CEP itself may serve as a vehicle to communicate to the ATCM the status of any non-native species, and promote appropriate collaboration and action.

### Policy Gaps

Recent experiences of non-native species eradication efforts have revealed several policy gaps that may merit further consideration by the CEP and the subsequent development of appropriate guidance. As noted above, the Protocol does allow pesticide use for scientific, medical or hygiene purposes (Annex III), but whether these include eradication of non-native species has not been formally addressed. The potential prohibition of pesticide use in the natural environment removes the main tool used elsewhere in the world for the control of non-native species. Given that the issue of non-native species introductions to Antarctica was not widely considered when the Protocol was being drafted prior to its agreement in 1991, any need for pesticides to enable species eradication may not have been considered. Further clarity from the CEP on the practical application of the information in the Protocol on pesticide use could reduce the risk of conflicting interpretations by Parties.

Annex II (Article 4, Para 5) to the Protocol states that any species not native to the Antarctic Treaty area that is introduced without a permit ‘shall be removed or disposed of whenever feasible, unless the removal or disposal would result in a greater adverse environmental impact’. So far, Parties have yet to attempt the eradication of any non-native invertebrate in the natural environment, and guidance on how to assess whether eradication of a non-native species would result in ‘greater adverse environmental impact’ is currently lacking. Although a series of studies have assessed the distribution and abundance of the introduced non-native midge *Eretmoptera murphyi* on Signy Island since c. 2008 (Hughes and Worland 2010; Hughes et al. 2013; Bartlett et al. 2019), the UK has decided not to undertake an eradication attempt due to the lack of an effective eradication methodology and the likely substantial impact upon one of the largest and richest areas of terrestrial habitat remaining on the island (Hughes and Worland 2010). This area is already subject to active protection measures against the impacts of the novel and anthropogenically rooted expansion of Antarctic fur seal presence on the island, which has seriously damaged or entirely destroyed a large proportion of the island’s previously rich terrestrial vegetation (Smith 1988; Hodgson et al. 1998; Waluda et al. 2010; Favero-Longo et al. 2011; Cannone et al. 2016, 2017). In hindsight, removal of the soil in the c. 1 m^2^ colonised area, when discovered in the early 1980s up to 20 years after the species’ presumed introduction (Burn 1982; Hughes and Worland 2010; Bartlett et al. 2019), could have resolved the issue with relatively little effort, and should serve as a lesson for those addressing future non-native invertebrate introductions. The potential for this midge to disperse further on the island and to colonise large areas of the Antarctic Peninsula and beyond (Hughes et al. 2013; Bartlett et al. 2019; Pertierra et al. 2019) only stands to highlight the error of not undertaking an early eradication effort when the midge was originally discovered. The provision of guidance by the CEP on the level of acceptable environmental damage caused by an eradication attempt and the range of endorsed methodologies available may facilitate more rapid response by Parties when faced with discoveries of established non-native species in the future. Such information may also help Parties complete the mandatory environmental impact assessment (EIA, see Annex I to the Protocol) process before commencing any eradication or control activities.

Finally, CEP guidance may also include information on which introduction events may trigger unilateral, multilateral or all Treaty Party responses. Sharing of information and receiving early warming of expanding non-native species distributions may allow Parties to amend their practices accordingly, or join in international efforts to monitor, control or eradicate the species as coordinated by COMNAP, CEP or any locally formed management groups (see Table 3).

### Science Needs

Should *T. maculipennis* be able to complete its life cycle in the Antarctic natural environment, as currently seems likely, answering several research questions will help inform subsequent management and control. For example, it would be useful to identify areas at high risk of colonization (including within local ASPAs), potentially by highlighting on a map important bird and seal concentrations (i.e., with high organic input; cf. Bokhorst et al. 2019) and areas with a large extent of vegetation (decaying plant material source), both of which provide habitat and food sources for *T. maculipennis*. Furthermore, with the increasing risk of non-native species colonizing Antarctic terrestrial habitats, there is an urgent need to assess the rate (and mechanisms, i.e., natural and/or anthropogenic) at which known successful invaders can extend their distribution and their preference for available habitats to best forecast other areas that may be at risk (cf. Bartlett et al. 2021). It is perhaps pertinent to note here that no instances of natural colonization events of terrestrial biota have been reported within what is now the Antarctic Treaty area throughout the history of human contact with this region, while only two putative instances (neither formally confirmed) have been proposed in the sub-Antarctic islands (the latter relative to over 200 anthropogenically assisted introduction events; Frenot et al. 2005). Thus, the overwhelming importance of anthropogenic influence in this matter is undeniable. Finally, it is essential to quantify the impacts of *T. maculipennis* on native biological communities (for example, the consequence of non-native *Eretmoptera murphyi* presence in peat soils on Signy Island results in up to almost an order of magnitude increase in nutrient turnover and nitrogen release (Hughes et al. 2013; Bartlett 2019)). The clear understanding of non-native species impacts is essential when assessing whether eradication attempts are merited, particularly given the environmental impacts that such actions might generate.

### Development of Monitoring and Eradication Programmes

In light of the CEP’s request that ‘coordinated standardised monitoring and eradication programmes’ are developed to effectively control the spread of *T. maculipennis*, we make the following observations. Data on *T. maculipennis* population trends throughout the year on stations and in the natural environment and the results of control measures using UV traps, combined with genetic analyses, will provide an improved picture of the scale of the problem and the means to address it. Practical management measures to reduce population sizes and restrict further dispersal should continue to be implemented and expanded (see Table 3). The data available to date suggest that the species has likely spread beyond the confines of research stations on King George Island. If monitoring carried out regularly confirms colonization of natural ecosystems, the probability of any eradication attempt being successful may be low and the impact on ecosystem processes high. Therefore, monitoring should be implemented, at a minimum, with regular inspection of traps installed in all stations present on King George Island and also in stations located on other regional islands. An additional benefit of installing traps in multiple stations is that it may give early warning of the import of other species, as well as an objective record of which species are likely to be regularly imported and persist synanthropically in station buildings, about which surprisingly little information is currently available.

A key lesson from the current situation is that considerable effort would have been saved if adequate biosecurity measures had been put in place across all introduction pathways to prevent the initial arrival of *T. maculipennis* (including, for example, thorough biosecurity checks of imported cargo and fresh foods, and the potential use of residual pesticides in high-risk areas within incoming vessels and aircraft). While there is merit in taking steps to reduce the dispersal and impact of this species, such efforts should not distract national operators and the tourism industry from implementing strict biosecurity measures to reduce the risk of further non-native species introductions. Given recent reports of non-native species in areas of high human activity and infrastructure across Antarctica (but in the Antarctic Peninsula region, in particular) further consideration of the mechanisms for communicating and coordinating multi-Party response actions should be a priority for policymakers.
